# Correction: David et al. Effects of Transient Loss of Vision on Head and Eye Movements during Visual Search in a Virtual Environment. *Brain Sci.* 2020, *10*, 841

**DOI:** 10.3390/brainsci11091215

**Published:** 2021-09-15

**Authors:** Erwan David, Julia Beitner, Melissa Le-Hoa Võ

**Affiliations:** Scene Grammar Lab, Department of Psychology, Theodor-W.-Adorno-Platz 6, Johann Wolfgang-Goethe-Universität, 60323 Frankfurt, Germany; beitner@psych.uni-frankfurt.de (J.B.); mlvo@psych.uni-frankfurt.de (M.L.-H.V.)

We wish to make the following correction to the published paper “Effects of Transient Loss of Vision on Head and Eye Movements during Visual Search in a Virtual Environment” [1].

We have identified a flaw in the implementation of a latency mitigation strategy for our gaze-contingent protocol written in Unity3D. As a result, the maximum latency is now estimated to be 30 ms instead of 15 ms, which should not affect any of the results originally published but should be noted for further reference.
